# True brachial artery aneurysm: A case report and review of literature

**DOI:** 10.1016/j.amsu.2020.06.011

**Published:** 2020-06-12

**Authors:** Youssef Shaban, Adel Elkbuli, Feargal Geraghty, Dessy Boneva, Mark McKenney, Jorge De La Portilla

**Affiliations:** Department of Surgery, Kendall Regional Medical Center, University of South Florida, Tampa, FL, USA

**Keywords:** True brachial artery aneurysm, Peripheral artery aneurysm, Aneurysm, Brachial artery

## Abstract

**Introduction:**

A true brachial artery aneurysm is a rare pathology with an incidence of 0.17% of all peripheral artery aneurysms. This pathology can manifest devastating complications if overlooked, however, a high index of suspicion coupled with a thorough history and physical allows easy diagnosis. We present a rare case of the oldest documented patient with a true brachial artery aneurysm with idiopathic etiology.

**Presentation of case:**

An 83-year-old gentleman presented with left upper extremity pain, erythema, and swelling for 1 week. He denied trauma to the area. Examination revealed a pulsatile mass of the antecubital fossa and decreased distal pulses. Imaging illustrated a 9mm aneurysm of the brachial artery with stenosis of the radial artery and non-enhancement of the origin of the ulnar artery. The patient underwent a brachial aneurysm excision, radial and ulnar embolectomy, and brachial to ulnar and radial artery bypass. Postoperatively, palpable pulses were appreciated in the radial and ulnar arteries. Pathology demonstrated a true aneurysm. The patient's postoperative course was uneventful and follow-up 6 months later revealed normal perfusion.

**Discussion:**

This case highlights the importance of maintaining a high index of suspicion coupled with a thorough history and physical examination when encountering neurovascular complaints of the upper extremities. Operative intervention even in asymptomatic patients is warranted due to a high complication rate of 33%.

**Conclusion:**

More research into the pathophysiology of this rare pathology is needed to further understand, prevent, or mitigate its complications.

## Highlights

• Upper extremity peripheral aneurysms account for less than 1% of all peripheral artery aneurysms with 0.5% involving the brachial artery and 0.17% being true aneurysms.
• Clinicians must maintain a high index of suspicion coupled with a thorough history/ physical examination when encountering neurovascular complaints of the upper extremities.
• The most widely accepted intervention based on data/expert opinion includes prompt diagnosis and operative resection of the aneurysm with interposition vein grafting.

## 1 Introduction

Brachial artery aneurysms comprise a rare group of vascular pathology with potentially devastating complications if missed but can easily be diagnosed with a high index of suspicion coupled with a thorough history and physical examination [1]. The vast majority are false or pseudoaneurysms compared to true aneurysms consisting of all 3 layers of the arterial wall. Compared to lower extremity aneurysms, upper extremity peripheral aneurysms are much less common and account for less than 1% of all peripheral artery aneurysms with 0.5% involving the brachial artery and only 0.17% being true aneurysms [1,2]. The etiology of false brachial artery aneurysms encompass trauma, iatrogenic, drug abuse, bacterial endocarditis, and mycotic lesions [[1], [2], [3], [4], [5]]. The increasing prevalence of invasive procedures such as arterial lines, dialysis access, and cardiac catheterizations via the upper extremity, attribute to brachial artery pseudoaneurysms being encountered more frequently [5]. On the other hand, the etiology of true aneurysms encompass atherosclerotic, genetic disease such as neurofibromatosis or vasculitides, especially Kawasaki's syndrome or Buerger's disease. In addition, previous surgery such as an arteriovenous fistula for dialysis and finally idiopathic pathology are other potential sources for this process [[1], [2], [3], [4], [5], [6]]. Patients with brachial artery aneurysms frequently are symptomatic and present with a palpable mass, pain/paresthesia, or acute limb ischemia due to thromboembolic sequela. Initially, asymptomatic lesions convert to be symptomatic in about 33% of cases [7]. This work has been reported in line with the SCARE criteria [8].

**Case Presentation:** An 83-year-old gentleman with a history of hypertension presented to our hospital with left upper extremity pain, erythema, and swelling for 1 week duration. The patient also endorsed decreased left hand grip strength. The patient denied any history of trauma to the area, smoking history, or family history of vasculitis or genetic diseases. On physical examination there was a moderate pulsatile mass at the left antecubital fossa of the volar aspect of the proximal forearm. This area had mild erythema with slight tenderness. The patient exhibited decreased left grip strength along with decreased radial and ulnar pulses compared to the right.

**Fig. 1:**
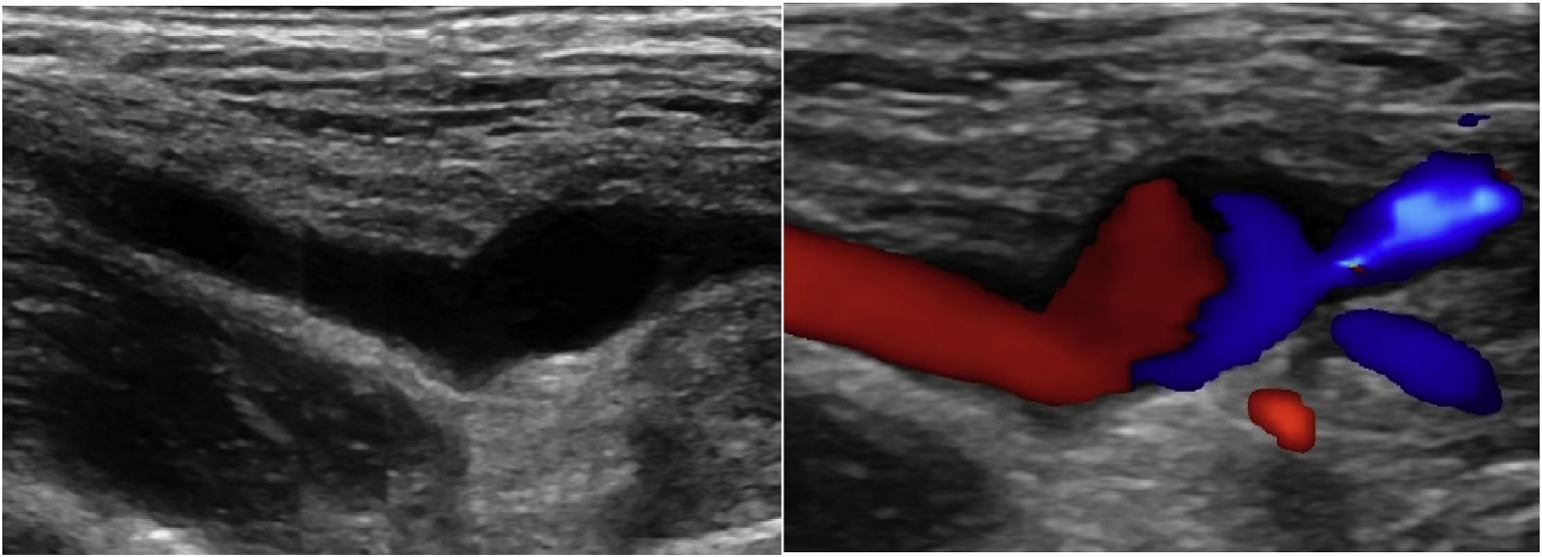
Ultrasound and Doppler images of the left upper extremity which shows fusiform aneurysmal dilatation of the brachial artery with turbulent flow.

**Fig. 2:**
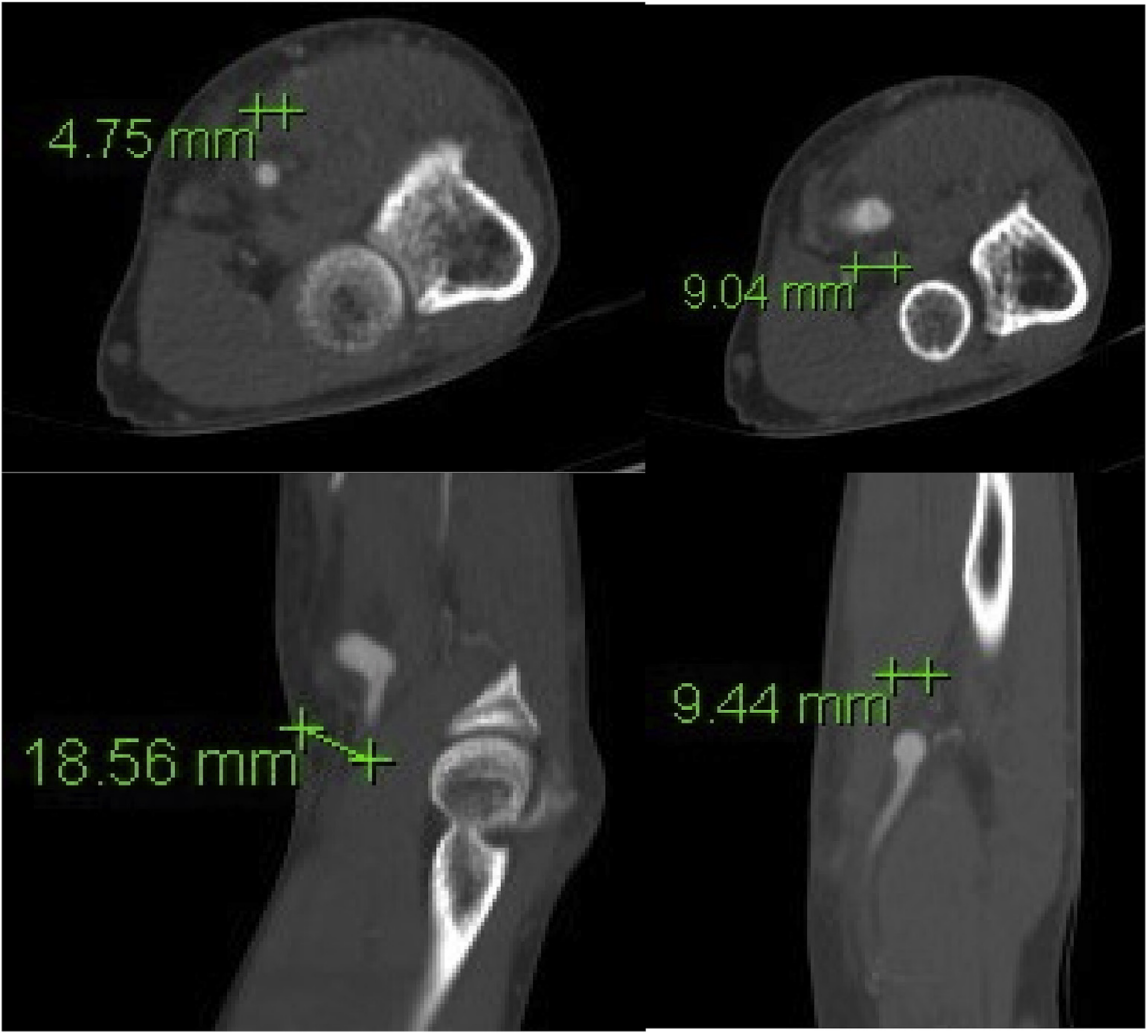
Axial CT angiogram images of the left upper extremity with a normal caliber brachial artery and 9 mm aneurysm of the brachial artery just distal to this at the level of the elbow (top row). Coronal and sagittal CT angiogram of the brachial artery aneurysm (bottom row).

**Fig. 3:**
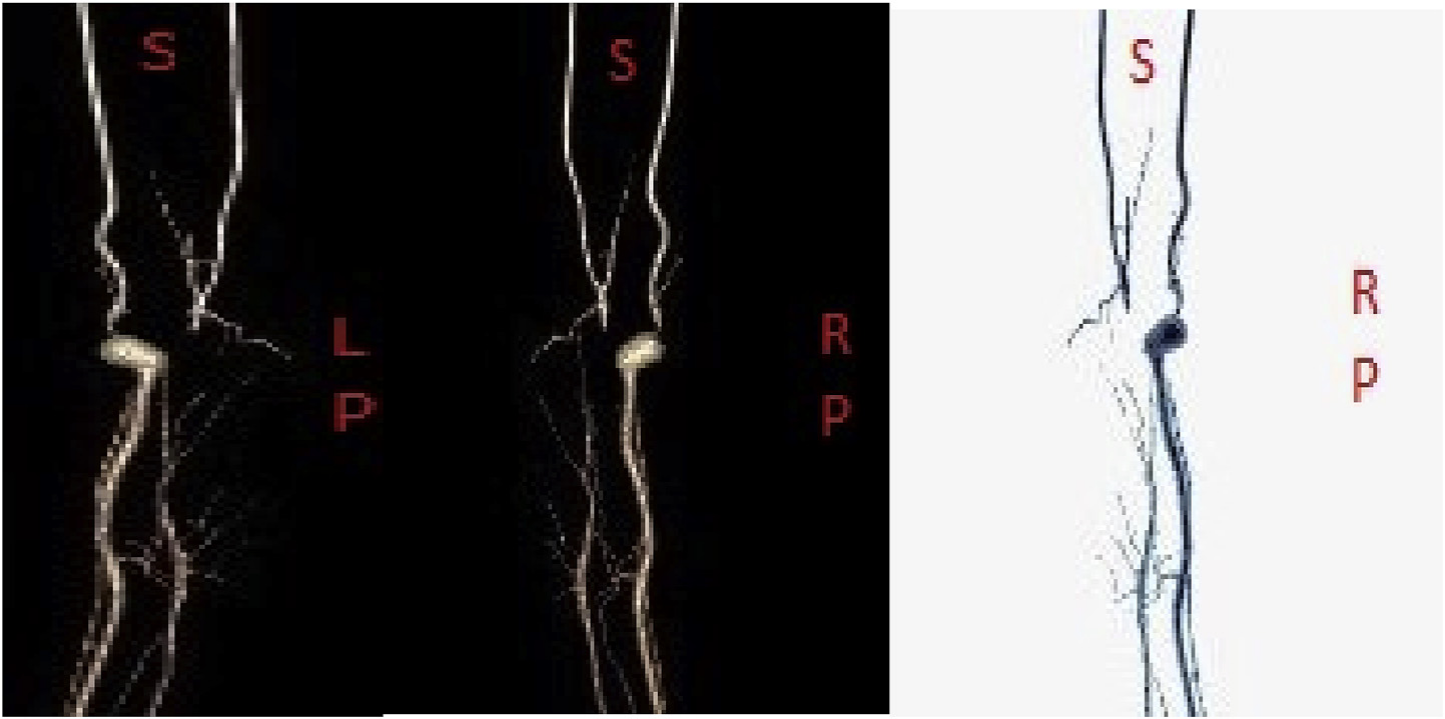
Reconstruction images of the CT angiogram illustrating a 9 mm aneurysm of the brachial artery at the level of the elbow, proximal to the bifurcation with severe stenosis at the origin of the radial artery and non-enhancement of the origin of the ulnar artery with well opacified distal branches. (S- superior, LP- left posterior, RP- right posterior).

**Fig. 4:**
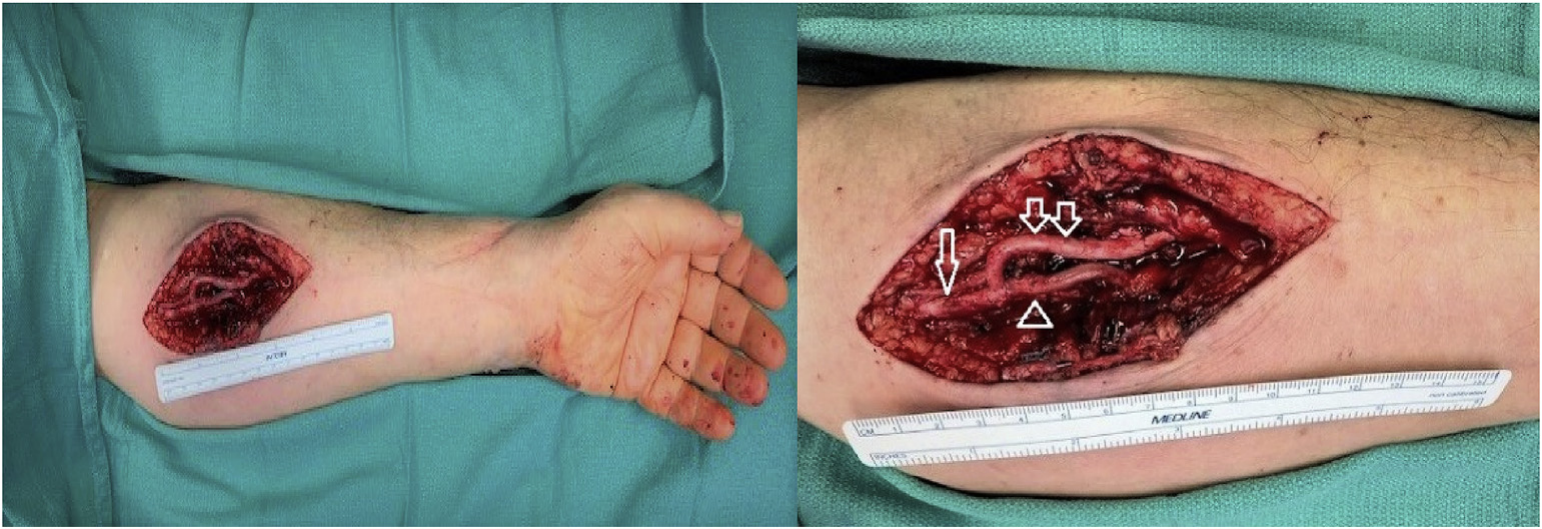
Intraoperative image illustrating a brachial artery to ulnar artery end-to-end bypass, and brachial to radial artery end-to-side anastomosis with reverse saphenous vein graft harvested from the left lower extremity. Arrow: brachial artery. Double arrows: radial artery. Arrow head: ulnar artery.

On workup, the patient had Doppler ultrasound imaging of the left upper extremity which showed a fusiform aneurysmal dilatation of the brachial artery with turbulent flow (Fig. 1). A computed tomography angiogram (CTA) illustrated a 9 mm aneurysm of the brachial artery at the level of the elbow, proximal to the bifurcation with severe stenosis at the origin of the radial artery. Additionally, there was non-enhancement of the origin of the ulnar artery but well opacified distal branches (Fig. 2, Fig. 3). The patient was diagnosed with a symptomatic brachial artery aneurysm versus pseudoaneurysm and was scheduled for surgery. Intraoperatively, we identified a brachial aneurysm just proximal to the bifurcation of the radial and ulnar arteries. The proximal end of the ulnar artery had no flow indicating an occlusion. There was no evidence of compression or entrapment of the vessels distal to the aneurysm. The patient underwent a left brachial artery aneurysm ligation and excision followed by a brachial to ulnar artery end-to-end bypass, and brachial to radial artery end-to-side anastomosis with reverse saphenous vein graft harvested from the left lower extremity (Fig. 4). Prior to completion an embolectomy with a Fogarty catheter #3 of the radial and ulnar arteries was carried out.

**Fig. 5:**
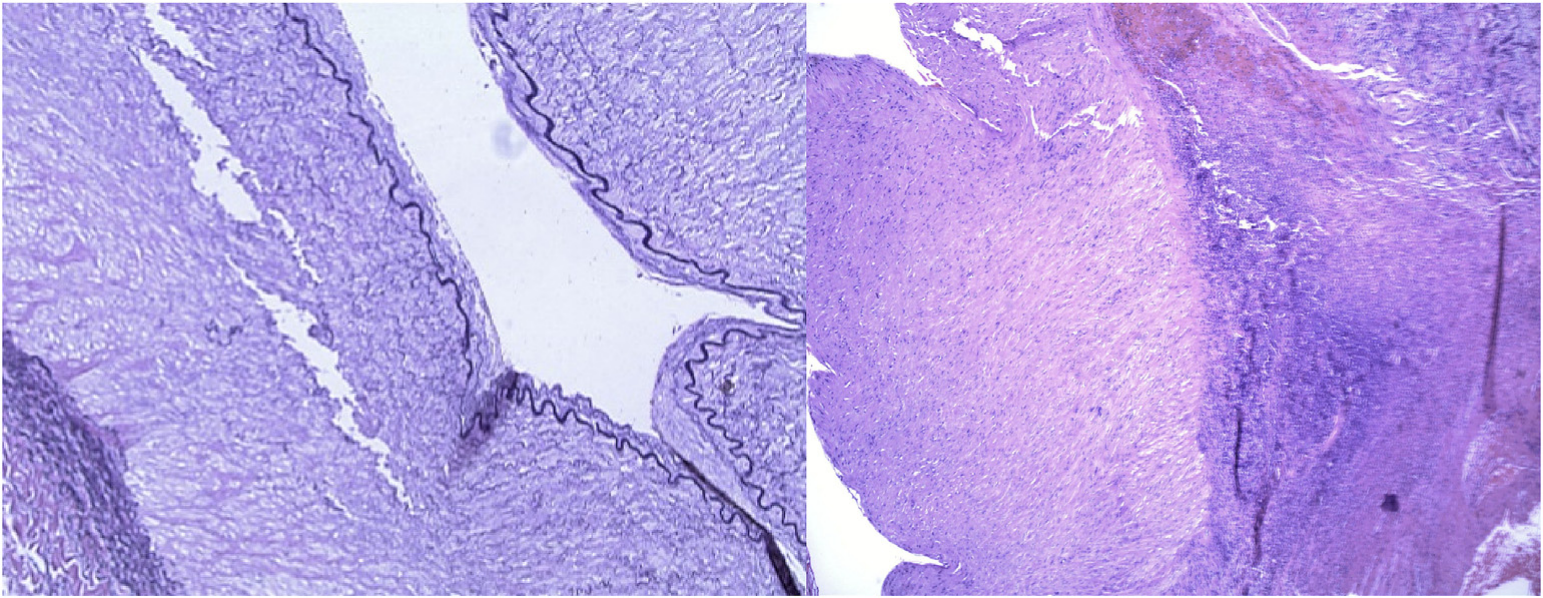
(Left) Histopathological analysis of aneurysmal specimen with an elastin stain denoted by the black line illustrating intact elastic lamina of the brachial artery surrounded by hyaline degeneration consistent with a true brachial artery aneurysm (40x magnification).(Right) Hematoxylin and eosin stained tissue section showing replacement of the tunica media by hyalinized connective tissue consistent with aneurysmal degeneration (40x magnification).

The surgery was successful with good flow through the anastomosed vessels. Palpable pulses were appreciated in the radial and ulnar arteries as well as Doppler signals in the palmar arch. The hand was noted to be well perfused with adequate capillary refill. Histopathological tissue analysis was consistent with a true aneurysm (Fig. 5). The patient's postoperative course was uneventful, and he was discharged home on the second postoperative day. At follow-up 6 months later, the patient had normal left hand function and perfusion.

## 2 Discussion

Upper extremity peripheral artery aneurysms are unusual but the vast majority of cases can be diagnosed with a high index of suspicion coupled with physical examination showing a pulsatile mass of the arm. Definitive diagnosis can be confirmed with Doppler ultrasonography, CTA, magnetic resonance angiography (MRA), or selective angiography [9].

Missed or delayed diagnosis carries potentially devastating morbidity including limb loss and life-threatening complications. There are no randomized control trials available in the literature regarding treatment, however, the most widely accepted intervention based on retrospective data and expert opinion includes prompt diagnosis and operative resection of the aneurysm with interposition vein grafting. This has been shown to have excellent outcomes with resolution of symptoms by removing the mass compressing local structures, minimal morbidity, and adequate long-term results [2,9,10]. As presented in our case, the patient had complete resolution of the initial complaint of decreased handgrip and a palpable radial and ulnar pulses post-operatively. Recently published novel endovascular treatment modalities have also been described particularly in high-risk surgical patients with pseudoaneurysms.

This pathology happens too infrequently for any single institution or surgeon to accumulate enough cases for meaningful statistical analysis. Most of the data published are case reports, surgeon experience, and retrospective analysis of hospital specific outcomes.

The Mayo clinic published the largest reported series of true upper extremity aneurysms distal to the axillary artery over a 20-year period, which only included 12 patients further supporting to the rarity of this disease. The most common presentation was a mass (67%), followed by pain or paresthesia (33%), and thromboembolic complications (25%). Interestingly, 33% of the asymptomatic patients who were followed for a mean of 6 years converted to symptomatic and required surgery. Authors concluded that due to the low morbidity of surgery, coupled with a relatively high and unpredictable conversion rate from asymptomatic to symptomatic, operative repair with excision and revascularization should be routinely performed [7].

In a comprehensive review article regarding peripheral aneurysms, Dawson et al. acknowledges that at the present time it is currently impossible to predict which asymptomatic aneurysms are likely to develop complications such as rupture or embolic ischemia. Authors emphasize that patients who undergo surveillance instead of treatment for asymptomatic brachial artery aneurysms carry a relatively high incidence rate for complications. Investigators also underscore the low morbidity associated with surgery and conclude all upper extremity aneurysms should be repaired regardless of symptoms, supporting the Mayo Clinic's conclusion [11].

In a review of 3 large-diameter true brachial artery aneurysms extending to the axillary zone, Senarslan et al. describes successful management by conventional surgery. Authors suggests the optimal treatment modality for brachial artery aneurysms is resection of the aneurysmal segment with subsequent interposition of the vein graft. Investigators emphasize several advantages of surgery including: removing the compression to the surrounding tissues, reestablishing distal circulation, low morbidity and mortality, and good long-term patency. Senarslan et al. acknowledges that newer endovascular methods may be an alternative therapeutic option, however, in their cases this was not possible due to unsuitable anatomy for interventional methods. Investigators acknowledge potential endovascular limitations depending on the type of aneurysm sac, intraluminal thrombosis, and the mobility of aneurysmal artery segments which may lead to stent fracture with flexion of the arm [2].

After conducting an extensive PubMed search of the English literature using the key words, “true brachial artery aneurysm” or “true brachial aneurysm”, 85 publications were identified with only 41 meeting the criteria of dealing with true brachial artery aneurysms. This included 80 cases with ages ranging from 9 months to 81 years with a mean age of 52. The most common etiology was previous arteriovenous fistula for dialysis and the most common presentation was symptomatic arm pain. It would appear that our patient is the oldest documented case of a true brachial artery aneurysm at 83 years old. It is well known that the prevalence of abdominal aortic aneurysms increase with age and major risk factors include an age older than 65 years, male gender, family history, and smoking habit [12]. Our patient meets two of the above risk factors being a male and 84 years old.

As described by Senarslan et al. true aneurysms are often atherosclerotic and degenerative in nature. Investigators recently published a report on 3 cases of large true brachial and axillary artery aneurysms that were successfully treated in a similar fashion as ours. Two of the cases included two of the oldest documented patients with a true brachial artery aneurysm being 78 and 81 years old, however, the authors did not elucidate further on the impact of the patients’ age [2].

In a retrospective observational single center study conducted in France over a 20 year period Fendri et al. analyzed 5 patients with true brachial artery aneurysm after arteriovenous fistula creation. Investigators also conducted an extensive literature review between 1996 and 2015 with 21 published cases (17 case reports, 3 series, and 1 review). The mean age among their patient cohort was 48.6 years for brachial artery aneurysms (range 37–76). The time spread after fistula creation and aneurysmal diagnosis was about 20.6 years (range 18–25) in their study and 20.5 years (range 9–29) in the literature. Authors concluded that the donor artery evolves toward true aneurysmal formation due to high flow and immunosuppression. Interestingly, authors suggest young age may act as a beneficial factor and delay aneurysmal formation [13].

## 3 Conclusion

We present a rare case of the oldest documented patient with a true brachial artery aneurysm with an idiopathic etiology that was successfully treated surgically. This case highlights the importance of maintaining a high index of suspicion coupled with a thorough history and physical examination when encountering neurovascular complaints of the upper extremities. Operative intervention even in asymptomatic patients is warranted due to a high complication rate encountered for observation. More research into the pathophysiology of this rare pathology is needed to further understand, prevent, or mitigate its complications.

## Ethical approval

This report was conducted in compliance with ethical standards. Informed written consent has been obtained and all identifying information is omitted.

## Author contribution

Please specify the contribution of each author to the paper, e.g. study design, data collections, data analysis, writing. Others, who have contributed in other ways should be listed as contributors.

## Funding

This research did not receive any specific grant from funding agencies in the public, commercial, or not-for-profit sectors.

## Informed consent

Informed written consent has been obtained and all identifying information is omitted.

## Registration of research studies

This is a case report study.

## Provenance and peer review

Not commissioned, externally peer reviewe

## Guarantor

The Guarantor is the one or more people who accept full responsibility for the work and/or the conduct of the study, had access to the data, and controlled the decision to publish.

## Acknowledgements

d.

## Declaration of competing interest

No conflicts of interest.
